# Anthropometric Status, Menstrual Health, and Psychosocial Correlates Among 4251 Adolescent Girls in Kazakhstan: A School-Based Cross-Sectional Study

**DOI:** 10.3390/healthcare14182918

**Published:** 2026-09-09

**Authors:** Ardak Ayazbekov, Aigul Terlikbayeva, Saken Khaidarov, Gulzhan Baigazieva, Zhaysan Imanbaeva, Aliya Aimbetova, Almagul Kurmanova, Damilya Salimbayeva

**Affiliations:** 1 Scientific Center of Obstetrics, Gynecology and Perinatology, 125 Dostyk Ave., 050010 Almaty, Kazakhstanguljan73@mail.ru (G.B.); zhaisan@mail.ru (Z.I.);; 2 Department of Molecular Biology and Medical Genetics, Asfendiyarov Kazakh National Medical University, 37A Zheltoksan Street, 050004 Almaty, Kazakhstan; 3 Faculty of Medicine and Healthcare, Al-Farabi Kazakh National University, 71 Al-Farabi Ave., 050040 Almaty, Kazakhstan

**Keywords:** adolescent health, menstrual health, dysmenorrhea, body mass index, underweight, psychosocial stress, nutrition, school health services, cross-sectional studies, Kazakhstan

## Abstract

Background/Objectives: Menstrual health in adolescence is influenced by nutritional, developmental, and psychosocial factors, yet integrated school-based evidence from Central Asia is limited. We assessed recorded anthropometric, menstrual/reproductive, and psychosocial indicators among adolescent girls in southern Kazakhstan and evaluated factors associated with a prespecified composite menstrual/reproductive outcome. Methods: We conducted a cross-sectional secondary analysis of a non-public school health screening database containing 5197 participant records from 2025. After removal of 946 exact duplicate copies, 4251 unique records of girls aged 12–18 years remained. We present prevalence estimates with Wilson 95% confidence intervals (CIs). We estimated adjusted prevalence ratios (aPRs) using modified Poisson regression with robust standard errors clustered by recorded school field. Results: Mean age was 15.03 ± 1.23 years and mean BMI was 20.36 ± 3.36 kg/m^2^. The source form used fixed adult BMI categories; these are reported descriptively and are not interpreted as pediatric nutritional diagnoses. At least one non-reference menstrual or reproductive indicator was recorded in 24.1% of participants; severe menstrual pain and stress were each recorded in 6.9%. Stress was associated with any menstrual/reproductive abnormality (aPR 2.05, 95% CI 1.65–2.54) and with severe menstrual pain (aPR 3.27, 95% CI 2.39–4.46). Unsatisfactory nutrition was also associated with the composite outcome (aPR 1.64, 95% CI 1.35–2.00). Conclusions: Recorded menstrual/reproductive concerns were frequent in this screened sample, and stress showed consistent associations with menstrual outcomes. Because anthropometric categories were based on adult cut points and several screening items were not validated instruments, interpret the findings as descriptive and hypothesis-generating rather than as population prevalence or causal estimates.

## 1. Introduction

Adolescence is a distinct developmental period during which reproductive maturation, body composition, and psychosocial functioning change rapidly [1,2]. Menarche and subsequent cycle maturation occur alongside changes in energy balance and adiposity. Low energy availability and reduced fat mass can delay menarche and disrupt hypothalamic-pituitary-ovarian function, whereas excess adiposity is associated with earlier pubertal timing and a greater likelihood of oligomenorrhea and hyperandrogenic features [3,4,5]. These pathways provide a biological rationale for examining anthropometric status together with menstrual health rather than as unrelated screening domains.

Menstrual symptoms are common during adolescence and can affect school attendance and daily functioning. Dysmenorrhea has been reported in a large proportion of adolescent and young adult populations, while irregular cycles and heavy bleeding may reflect normal early post-menarcheal maturation or clinically relevant conditions requiring evaluation [6,7,8,9,10]. For this reason, menstrual characteristics are increasingly considered an important component of adolescent health assessment [7].

Psychosocial and behavioral factors may intersect with these reproductive processes. Perceived stress has been associated with menstrual irregularity and greater menstrual pain in student and occupational cohorts [11,12,13,14], and dietary patterns have also been linked to menstrual symptoms [15,16]. These relationships may be bidirectional: stress can influence neuroendocrine regulation, while painful or unpredictable menstruation can itself contribute to distress. Consequently, interpret cross-sectional associations as co-occurrence rather than evidence of directionality.

Access to age-appropriate menstrual and reproductive health information and services is not uniform across health systems. Gaps have been described particularly in low- and middle-income settings and in systems where adolescent, pediatric, school-health, and reproductive-health services are weakly integrated [17,18,19,20]. We therefore avoid assuming that all adolescents universally ‘fall between’ pediatric and obstetric care; the relevant concern is whether adolescents in a given setting receive systematic, developmentally appropriate assessment and referral.

Evidence linking anthropometric, menstrual, and psychosocial indicators within the same large school-based sample remains limited in Central Asia. What has been published on adolescent health in Kazakhstan comes mainly from broad public-health assessments and national household surveys [21,22], and comparative burden figures for this age band rest largely on modelled global estimates rather than on direct measurement [23]. The present study analyzes an investigator-maintained school health screening dataset collected in the Turkistan region of Kazakhstan during 2025. Its value lies in the simultaneous recording of anthropometric measurements, menstrual/reproductive indicators, nutrition, stress, and social wellbeing in an understudied population. The main evidence gap is therefore not simply the prevalence of any single symptom, but the patterns of co-occurrence and adjusted associations across these domains.

The primary outcome was a prespecified composite of any non-reference menstrual/reproductive indicator (non-reference menarche, abnormal menstrual duration, abnormal menstrual flow, or severe menstrual pain). The primary hypothesis was that clinician-recorded stress and unsatisfactory nutrition would be associated with a higher prevalence of this composite outcome after adjustment for age, recorded body-mass category, and chronic disease. Secondary outcomes were severe menstrual pain and non-reference reproductive status; we hypothesized that stress would be positively associated with both and that severe menstrual pain would increase with age. We treated age-related patterns in the source-form BMI categories as secondary descriptive analyses rather than tests of pediatric nutritional diagnoses because the source form used fixed adult BMI cut points.

Accordingly, the study objectives were to (1) describe the recorded anthropometric, menstrual/reproductive, and psychosocial profile of the screened sample; (2) examine age-related variation in selected indicators; and (3) estimate adjusted cross-sectional prevalence ratios for the prespecified primary and secondary outcomes while accounting for clustering by the recorded school field.

## 2. Materials and Methods

### 2.1. Study Design and Setting

We performed a cross-sectional secondary analysis of an investigator-maintained, non-public school health screening database; it is not an open-access registry and has no public URL. We conducted screening visits in 2025 in secondary schools, lyceums, gymnasiums, and one boarding school in Turkistan, Kentau, and surrounding settlements in the Turkistan region, southern Kazakhstan. The analytic spreadsheet available for this study did not retain exact screening months and visit dates, so we cannot report them retrospectively without speculation. Examinations were performed by visiting multidisciplinary teams that included obstetrician-gynecologists. The database contained 198 distinct school-name strings; because school names were entered inconsistently, these strings cannot be assumed to represent 198 verified unique institutions, and the analytic file cannot be used to reconstruct an exact urban-versus-rural school count. Reporting follows the STROBE recommendations for cross-sectional studies [24].

### 2.2. Participants

Eligible participants were girls aged 12–18 years who were present at school on the day of the screening visit and whose parents or legal guardians provided written consent; adolescents provided assent. The study used all eligible records available in the screening database rather than a probability sample. Because there was no formal invitation list or population sampling frame, we cannot calculate a response rate or sampling fraction. The study size was therefore determined by the number of available eligible records rather than by an a priori power calculation. Records outside the 12–18-year age range and records lacking age, weight, height, or BMI were excluded from the relevant analyses.

### 2.3. Measurements and Source Variables

Height was measured by the field teams using a stadiometer and recorded to the nearest centimeter. Weight was measured in light clothing without shoes using the weighing device available to the screening team and was recorded to the nearest kilogram. The retrospective analytic file and accompanying source documentation did not retain the manufacturer, model, country of origin, calibration log, or device-specific metadata for the stadiometer or weighing device; these details therefore cannot be reconstructed reliably and are acknowledged as a measurement limitation. BMI was calculated as weight in kilograms divided by height in meters squared and stored to one decimal place. Recorded BMI values closely matched values recalculated from height and weight; the maximum absolute discrepancy was 0.05 kg/m^2^.

The screening form contained coded fields for chronic disease and diagnosis, body-mass category, reproductive health status, age at menarche, menstrual duration, menstrual flow, menstrual pain, sexual activity, stress, nutrition, and social wellbeing (Table A1). These were operational categories used by the screening program rather than a set of validated diagnostic scales. The source body-mass codes followed fixed adult BMI cut points; for adolescents, WHO BMI-for-age standards are the appropriate reference framework [25,26]. Exact age in months or date of birth was not available in the analytic file, preventing valid retrospective calculation of WHO 2007 BMI-for-age z-scores. Menstrual duration and symptom interpretation were considered in relation to adolescent menstrual guidance [7], while the broader concept of menstrual health was interpreted in accordance with contemporary definitions [20]. Menstrual blood loss was not quantified in milliliters; the source field recorded only categorical clinician-coded flow (moderate, scanty, heavy, or no menses). Stress, nutrition, social wellbeing, reproductive status, and menstrual pain were likewise clinician-coded source-form items and were not validated psychometric or diagnostic instruments.

### 2.4. Derived Indicators

Derived indicators were prespecified before analysis. The source-form underweight-range indicator corresponded to body-mass code 4; the BMI ≥ 25 indicator to codes 1–3; and the BMI ≥ 30 indicator to codes 2–3. These labels describe the source coding and are not pediatric diagnoses. We defined non-reference reproductive status as any source code other than ‘corresponds to age,’ and non-reference menarche as any category other than 11–13 years. Abnormal menstrual duration and abnormal menstrual flow were defined as any code outside the source reference category. Severe menstrual pain corresponded to the explicit severe-pain code. The primary composite outcome, ‘any menstrual/reproductive abnormality’, was positive when at least one of the following was present: non-reference menarche, abnormal duration, abnormal flow, or severe menstrual pain. Secondary outcomes were severe menstrual pain and non-reference reproductive status. An amenorrhea/no-menses indicator combined the corresponding source codes across menstrual duration, flow, and pain. The exploratory domain count was retained for descriptive purposes only and has no validated clinical interpretation.

### 2.5. Data Cleaning and Reconciliation

**Figure 1:**
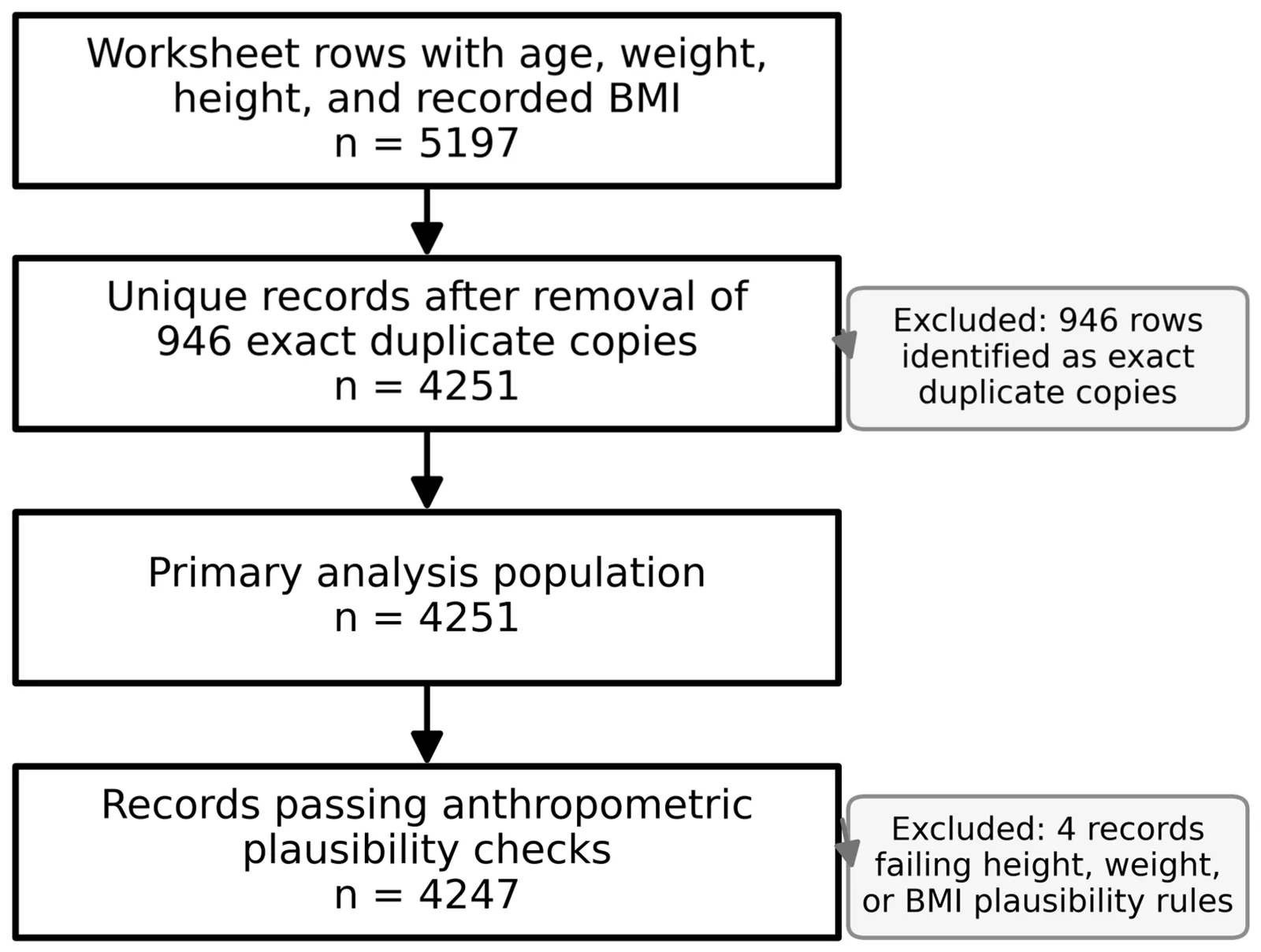
Participant-record flow through deduplication and anthropometric plausibility checks. The primary analytic dataset included 4251 unique records; four records were excluded only from anthropometric summaries because they failed prespecified plausibility criteria.

The worksheet contained 5215 non-empty rows, of which 5197 represented participant records, and 18 were summary rows. Data inspection identified a 943-row block (Excel rows 1086–2028) that was identical, cell for cell, to rows 2029–2971, together with three additional exact duplicate copies elsewhere in the worksheet. The block structure most strongly suggests a repeated copy/paste or duplicate batch import during manual spreadsheet consolidation, but no audit trail was available to establish the operational cause with certainty. We therefore report the duplication pattern without assigning an unverified cause. In total, we removed 946 exact duplicate copies while retaining the first occurrence, leaving 4251 unique records (Figure 1). Sensitivity analysis showed that deduplication changed key prevalence estimates by less than 0.7 percentage points (Section 3.7).

Anthropometric plausibility criteria were applied next: height 120–200 cm, weight 25–150 kg, and BMI 10–60 kg/m^2^. Four records failed at least one criterion and were excluded from anthropometric summaries, leaving 4247 records. One sexual-activity code and one social wellbeing code were outside the permitted coding range and were set to missing. Nineteen records indicated chronic disease without diagnosis text, and one included diagnosis text despite a negative chronic-disease code; these records were retained with the inconsistency flagged. Because source forms were not available for record-level adjudication, the exact causes of implausible or out-of-range values cannot be established. Plausible mechanisms include transcription, coding, or unit-entry errors during manual data transfer; this is treated as a data-quality limitation rather than assumed to be the confirmed cause.

### 2.6. Statistical Analysis

**Figure 2:**
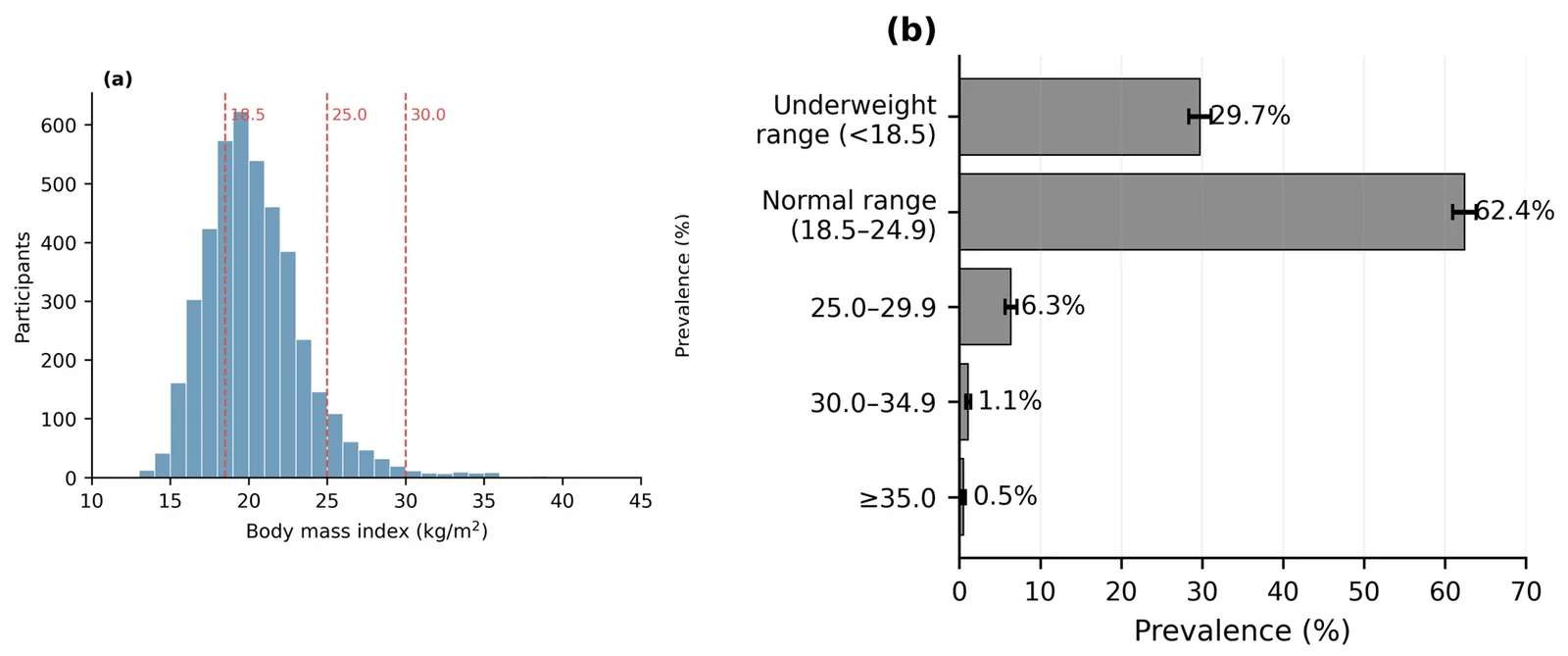
Body mass index among adolescent girls aged 12–18 years. (**a**) BMI distribution among 4247 records that passed anthropometric plausibility checks; vertical dashed lines indicate adult reference cut points at 18.5, 25.0, and 30.0 kg/m^2^. (**b**) Prevalence of recorded BMI categories with Wilson 95% CIs. The categories are descriptive because pediatric BMI-for-age references were not available in the source form.

Continuous variables are summarized using mean and standard deviation (SD) together with median, interquartile range (IQR), and range. Distributional form was evaluated using the observed distributions and graphical inspection; BMI was visibly right-skewed (Figure 2a). Because several continuous variables were non-Gaussian and age-group sizes were highly unequal, nonparametric comparisons were preferred rather than relying on formal normality tests, which can detect trivial deviations in very large samples. Binary variables are reported as counts and percentages with Wilson 95% confidence intervals (CIs) [27,28]. Differences across age groups were assessed using chi-square tests for categorical variables with bias-corrected Cramér’s V as the effect-size measure and Kruskal–Wallis tests for continuous variables.

The previously reported Spearman/phi correlation matrix was removed from the revised manuscript because individual observations were clustered within schools and an unclustered correlation analysis would not reflect that dependence structure. In addition, several pairwise correlations (for example, between source-form menarche and reproductive-status categories) did not materially advance the prespecified study objectives. The revised inferential analysis therefore focuses on school-clustered prevalence-ratio models.

Adjusted prevalence ratios (aPRs) were estimated using modified Poisson regression with a log link and robust sandwich standard errors clustered by the recorded school field [29,30]. This method estimates prevalence ratios directly and uses robust variance estimation to address variance misspecification when Poisson models are applied to binary outcomes. Covariates were specified a priori from the literature and the conceptual framework linking age, body composition, chronic disease, stress, and nutrition with menstrual function [4,5,6,7,8,9,10,11,12,13,14,15,16,31,32], rather than selected solely because of statistical significance. The main models included age, the two mutually exclusive source-form body-mass indicators (underweight range and BMI ≥ 25), chronic disease, stress, and unsatisfactory nutrition where permitted by the outcome definition. Models were interpreted as associational, not causal. Formal variance-inflation-factor output from the original analytic run was not preserved in the analysis archive; we therefore do not report retrospective VIF values that cannot be verified. This absence is acknowledged as a reporting limitation.

School-level variation was examined descriptively among recorded school-name strings contributing at least 30 unique records. Because the school field was not standardized, these analyses are exploratory and should not be interpreted as institutional rankings [33]. All statistical tests were two-sided, with *p* < 0.05 used as a conventional significance threshold; effect sizes and 95% CIs were emphasized in interpretation. Analyses were conducted in Python (version 3.14.7; Python Software Foundation, Beaverton, OR, USA) with the pandas, NumPy, SciPy, and statsmodels libraries. Version numbers for the individual libraries were not retained in the analysis archive, so the packages are named without them.

### 2.7. Ethical Approval

The program under which these data were collected was reviewed and approved by the Local Bioethics Committee of the Academy of Preventive Medicine, Almaty, Kazakhstan, on 21 May 2025 (protocol No. XIII-R). The committee confirmed that the project complied with international bioethical principles and Good Clinical Practice and that procedures for personal data protection and voluntary informed consent were in place. Written informed consent was obtained from parents or legal guardians, with assent from participating adolescents. Names were used only to identify exact duplicate rows and were removed before analysis; no participant-identifying information is presented in this article.

## 3. Results

### 3.1. Study Population

After exact deduplication, 4251 unique records were available for analysis. Participants were 12–18 years old, with a mean age of 15.03 ± 1.23 years; girls aged 14–16 years accounted for 77.4% of the sample (Table 1). Four records were excluded only from anthropometric summaries because of prespecified plausibility criteria, leaving 4247 records for height, weight, and BMI. Missingness was otherwise minimal for the reported source-form indicators: one sexual-activity code and one social wellbeing code were treated as missing.

### 3.2. Anthropometric Profile

The BMI distribution was right-skewed (Figure 2a). According to the fixed adult cut points embedded in the source screening form, 1263 girls (29.71%, 95% CI 28.36–31.10) were assigned to the underweight-range category, 2652 (62.39%) to the 18.5–24.9 category, 270 (6.35%) to 25.0–29.9, 45 (1.06%) to 30.0–34.9, and 21 (0.49%) to ≥35.0 (Table 2; Figure 2b). These percentages describe the source-form classification only. They should not be interpreted as valid pediatric prevalence estimates because exact age in months was unavailable and WHO BMI-for-age z-scores could not be reconstructed.

Within the source-form underweight-range category, median BMI was 17.3 kg/m^2^ (IQR 16.4–17.9), placing many observations near the adult 18.5 kg/m^2^ threshold. The strong age gradient in this recorded category is therefore particularly vulnerable to misclassification when using fixed adult cut points during adolescence.

### 3.3. Menstrual, Reproductive and Psychosocial Indicators

**Figure 3:**
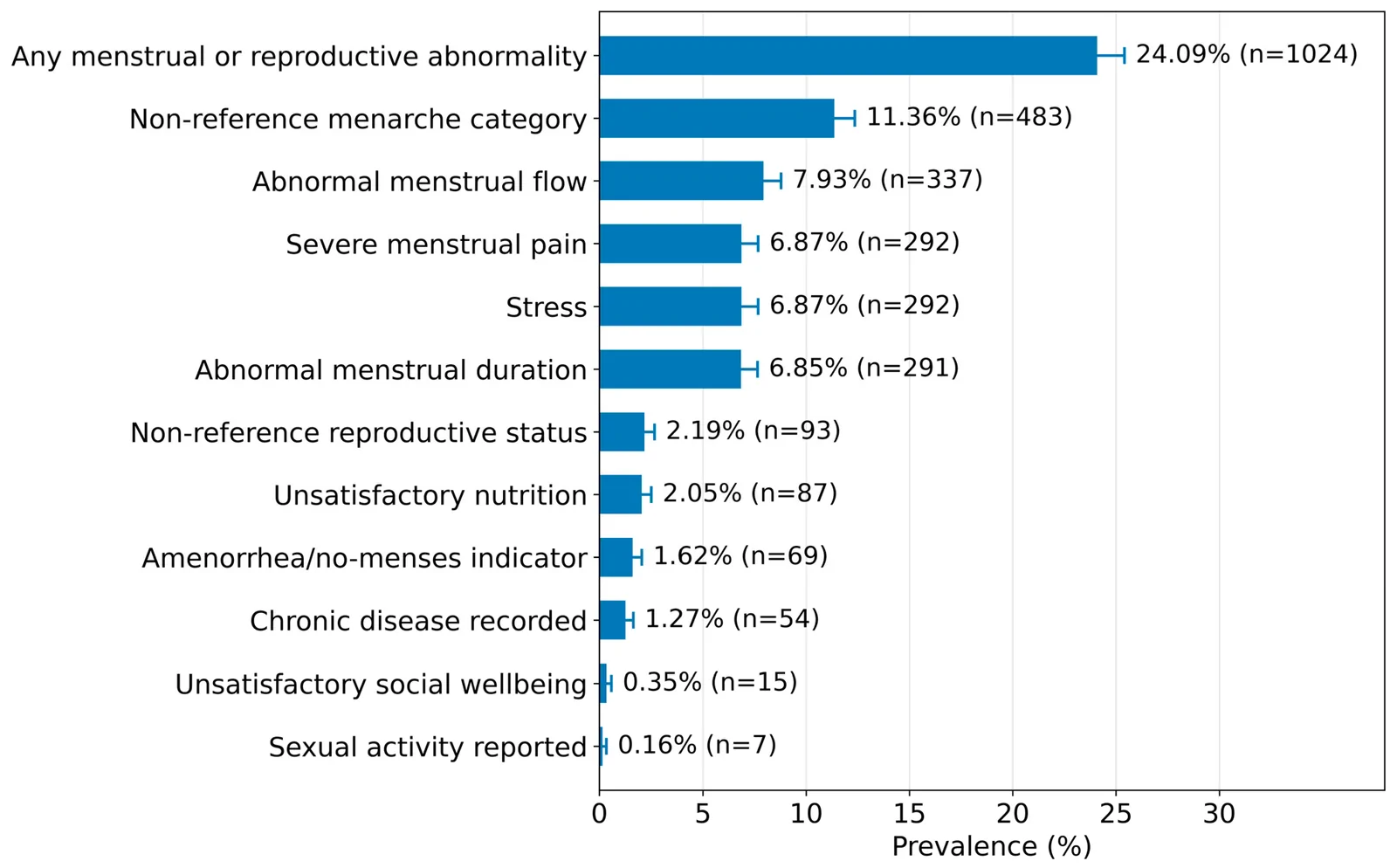
Prevalence of selected menstrual, reproductive, psychosocial, and chronic-health indicators. Error bars show Wilson 95% confidence intervals. Percentage labels and event counts are positioned beyond the confidence-interval whiskers to avoid overlap.

At least one non-reference menstrual or reproductive indicator was recorded in 1024 girls (24.09%, 95% CI 22.83–25.40). Non-reference menarche was the most frequent individual finding (483; 11.36%), followed by abnormal menstrual flow (337; 7.93%), severe menstrual pain (292; 6.87%), and abnormal menstrual duration (291; 6.85%). The amenorrhea/no-menses composite was present in 69 girls (1.62%), non-reference reproductive status in 93 (2.19%), and chronic disease in 54 (1.27%). Complete prevalence estimates are presented in Table 3 and Figure 3.

Among the psychosocial indicators, stress was recorded in 292 girls (6.87%), unsatisfactory nutrition in 87 (2.05%), and unsatisfactory social well-being in 15 of 4250 valid records (0.35%). Sexual activity was reported in seven girls (0.16%). Given the likely influence of reporting and social desirability factors on this item, sexual activity was not analyzed further.

### 3.4. Age-Related Patterns

**Figure 4:**
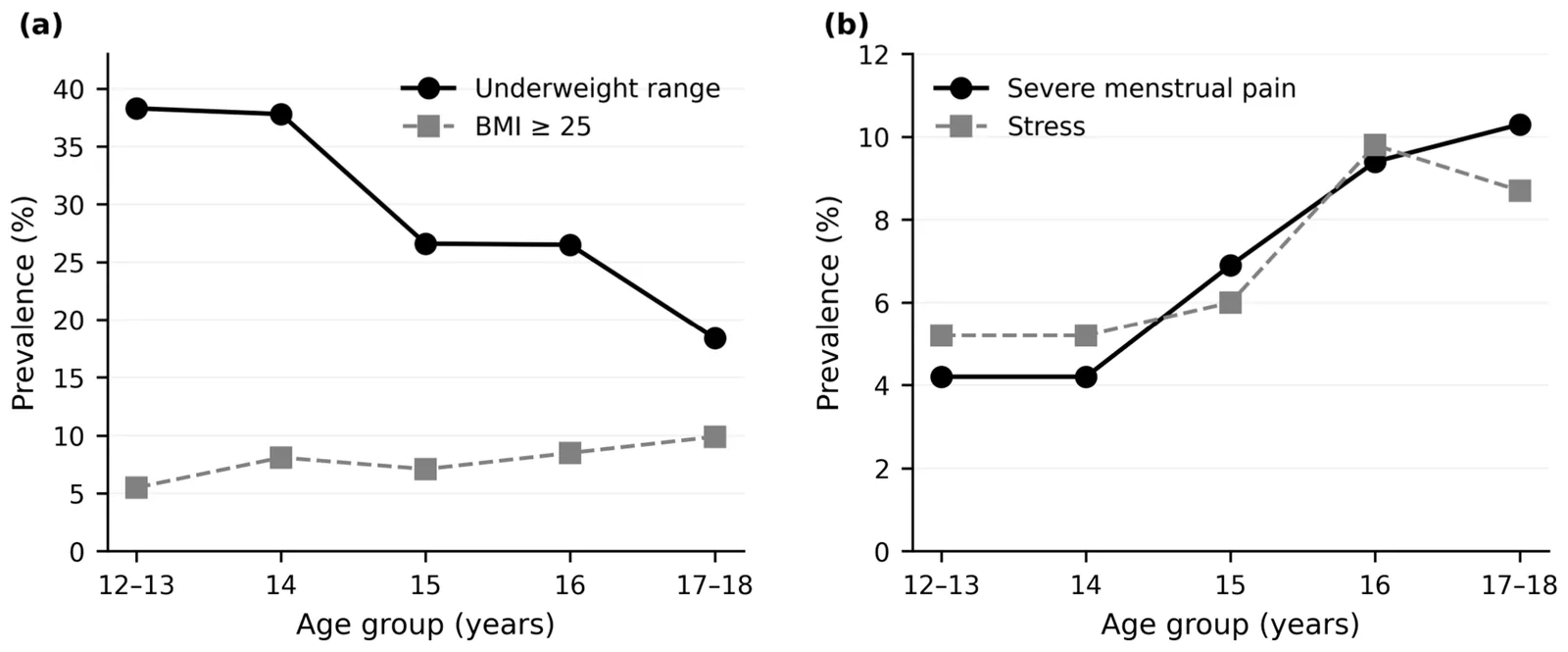
Age-related prevalence patterns. (**a**) Underweight-range and BMI ≥ 25 categories. (**b**) Severe menstrual pain and stress. Values represent the percentage of girls within each age group.

Age-related patterns differed across the recorded outcome domains (Table 4; Figure 4). The source-form underweight-range category decreased from 38.3% at ages 12–13 to 18.4% at ages 17–18 (χ^2^ = 96.34, *p* < 0.001; Cramér’s V = 0.147). Because this category uses a fixed adult threshold, the gradient cannot be interpreted as an age-specific change in pediatric underweight prevalence. The source-form BMI ≥ 25 category increased from 5.5% to 9.9%, although overall heterogeneity did not reach statistical significance (*p* = 0.095).

Severe menstrual pain increased from 4.2% in the youngest age group to 10.3% at ages 17–18 (χ^2^ = 36.54, *p* < 0.001). Stress showed a similar age-related pattern and peaked at age 16 (9.8%; χ^2^ = 23.29, *p* < 0.001). Non-reference menarche, non-reference reproductive status, and unsatisfactory nutrition did not differ significantly across age groups. Weight, height, BMI, and the exploratory domain count differed across age groups on Kruskal–Wallis testing (all *p* < 0.001).

### 3.5. Multivariable Models

**Figure 5:**
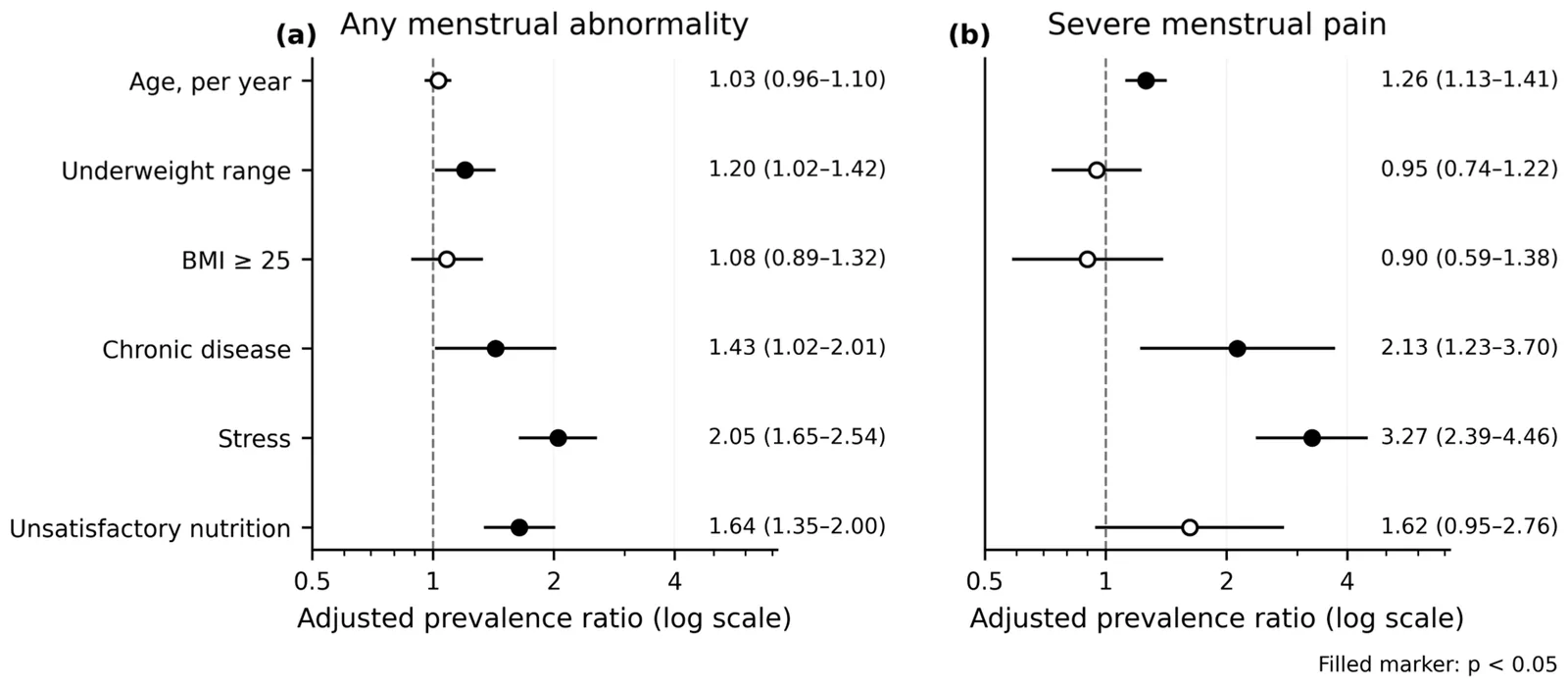
Adjusted prevalence ratios (aPRs) with school-clustered 95% CIs for (**a**) any menstrual abnormality and (**b**) severe menstrual pain. The vertical dashed line denotes the null value (aPR = 1.0). Filled markers indicate *p* < 0.05; open markers indicate *p* ≥ 0.05.

Adjusted prevalence ratios are summarized in Table 5 and Table 6 and visualized in Figure 5. For the primary composite outcome (1024 events), stress showed the largest adjusted association (aPR 2.05, 95% CI 1.65–2.54), followed by unsatisfactory nutrition (aPR 1.64, 95% CI 1.35–2.00), chronic disease (aPR 1.43, 95% CI 1.02–2.01), and the source-form underweight-range indicator (aPR 1.20, 95% CI 1.02–1.42). Age and the source-form BMI ≥ 25 indicator were not independently associated with the composite outcome.

For severe menstrual pain (292 events), stress remained the strongest correlate (aPR 3.27, 95% CI 2.39–4.46). Chronic disease was associated with approximately twice the prevalence (aPR 2.13, 95% CI 1.23–3.70), and each additional year of age was associated with a 26% higher prevalence (aPR 1.26, 95% CI 1.13–1.41). Neither body-mass category was independently associated with severe menstrual pain.

For non-reference reproductive status (93 events), stress was associated with higher prevalence (aPR 2.78, 95% CI 1.71–4.51). The estimate for underweight-range status was also elevated but imprecise and did not meet the conventional threshold for statistical significance (aPR 1.51, 95% CI 0.98–2.31; *p* = 0.059).

When stress was modeled as the outcome, unsatisfactory nutrition (aPR 5.97, 95% CI 3.79–9.40) and unsatisfactory social wellbeing (aPR 5.14, 95% CI 2.91–9.07) showed the largest associations. The social wellbeing estimate was based on only 15 events and should therefore be interpreted cautiously. The underweight-range category decreased with age (aPR 0.83 per year, 95% CI 0.79–0.88), whereas the BMI ≥ 25 category increased modestly with age (aPR 1.09 per year, 95% CI 1.00–1.19).

### 3.6. School-Level Variation

The database contained 198 distinct school-name strings, of which 31 contributed at least 30 unique records. Because school names were entered inconsistently, the number of strings cannot be equated with the number of unique participating schools, and the analytic file did not contain a standardized urban/rural school classification. Prevalence varied across the 31 higher-volume strings for both the source-form underweight-range category (χ^2^ = 109.27, df = 30, *p* < 0.001) and the composite menstrual/reproductive outcome (χ^2^ = 275.60, df = 30, *p* < 0.001). These differences may reflect true catchment heterogeneity, examiner effects, or inconsistent school labeling and should not be interpreted as school performance rankings.

### 3.7. Sensitivity Analyses

Repeating the main prevalence estimates using the raw file before duplicate removal changed each estimate by less than 0.7 percentage points (Table 7). Retaining the four anthropometrically implausible records increased mean BMI from 20.36 to 20.81 kg/m^2^ while leaving the median unchanged at 19.9 kg/m^2^, illustrating the mean’s sensitivity to a small number of extreme data-entry values and supporting the presentation of both mean and median summaries.

## 4. Discussion

Three findings are most defensible in this school-based sample of 4251 adolescent girls. First, a substantial proportion of records were assigned to the source-form underweight-range category, but this cannot be interpreted as a pediatric underweight prevalence because fixed adult BMI cut points were used. Second, approximately one quarter of participants had at least one non-reference menstrual/reproductive indicator, and clinician-recorded severe menstrual pain increased with age. Third, stress and unsatisfactory nutrition showed the most consistent adjusted associations with menstrual outcomes. These results support further study of the intersection between anthropometric, menstrual, and psychosocial domains, but they do not establish causal pathways or intervention effects.

### 4.1. Anthropometric Status and the Limits of Adult Cut Points

The source form assigned 29.7% of records to an underweight-range category based on the fixed adult threshold of 18.5 kg/m^2^. This figure should not be described as the prevalence of adolescent underweight. BMI changes with age during adolescence, and pediatric classification should use sex- and age-specific BMI-for-age standards [25,26]. The analytic dataset contained age only in completed years and did not include date of birth or exact age in months, which prevents valid retrospective calculation of WHO 2007 BMI-for-age z-scores. The observed decline in the source-form underweight-range category from 38.3% at ages 12–13 to 18.4% at ages 17–18 may therefore be driven partly, and potentially substantially, by the classification rule itself.

Regional literature indicates that adolescent thinness and increasing overweight can coexist in Central and South Asia [34,35,36,37]. However, the present study cannot quantify either condition using pediatric diagnostic criteria. We therefore retain the source-form categories only to characterize how the original screening program classified participants and to examine whether those recorded categories were associated with menstrual outcomes. Future data collection should retain date of birth or age in months and prospectively apply WHO BMI-for-age z-scores.

### 4.2. Menstrual Findings in Context

The composite menstrual/reproductive indicator was recorded in 24.1% of participants, but its components are source-form screening categories rather than validated diagnostic outcomes. The 6.9% frequency of clinician-recorded severe menstrual pain is much lower than the broader prevalence of dysmenorrhea reported in meta-analyses [6], which is unsurprising because the present field captured only a clinician-coded severe-pain category and did not use a validated pain scale. Menstrual flow was also qualitative rather than volumetrically measured. Consequently, these frequencies should be interpreted as the proportion of screened records meeting the program’s operational categories, not as population estimates of clinically defined menstrual disorders.

The increase in clinician-recorded severe menstrual pain from 4.2% at ages 12–13 to 10.3% at ages 17–18 is consistent with the increasing prominence of dysmenorrhea after ovulatory cycles become established [7,8,31], but the cross-sectional design and absence of gynecological age preclude a developmental trajectory analysis. Persistent or increasing pain remains clinically relevant because secondary causes such as endometriosis may require evaluation [7], but this study did not diagnose those conditions.

### 4.3. Stress and Nutrition

Stress was the most consistent psychosocial correlate, with higher adjusted prevalence of the primary composite outcome, severe menstrual pain, and non-reference reproductive status. These cross-sectional associations cannot determine temporal direction. Stress may influence menstrual function through neuroendocrine pathways [11,12,13,14,32], menstrual symptoms may increase distress, and shared clinician-reporting processes may inflate co-occurrence among subjective items. Using a single binary stress item further limits construct validity.

Unsatisfactory nutrition was also associated with the primary menstrual/reproductive composite. However, this source-form item was not based on dietary recall, food-frequency assessment, or a validated nutrition score, and laboratory measures such as hemoglobin or ferritin were unavailable. The result should therefore be interpreted as an association with a clinician-recorded nutrition concern, not evidence of a specific nutritional mechanism.

### 4.4. Variation Between Schools

Between-school-string variation was substantial, but its interpretation is limited by inconsistent school naming and the absence of standardized urban/rural, socioeconomic, and examiner identifiers. Some heterogeneity may reflect true catchment differences, while other variation may reflect data-entry or examiner effects. The analysis therefore uses school strings only to cluster robust standard errors and to describe heterogeneity, not to rank institutions.

### 4.5. Implications for School Health Services

The findings suggest that anthropometric, menstrual, and psychosocial domains can co-occur within school health screening and merit joint consideration in future research. However, this study did not evaluate a screening intervention or downstream clinical outcomes. It therefore cannot show that integrating these assessments will improve health, attendance, referral, or treatment outcomes. Prospective implementation studies are needed to test whether integrated, validated screening protocols improve identification and care compared with existing practice [19,20,38].

Future screening rounds could improve measurement quality by recording exact age in months or date of birth and applying WHO BMI-for-age standards [25]; using validated measures of menstrual pain, cycle characteristics, psychosocial stress, and diet [7,10,20]; documenting device model and calibration status; standardizing school and examiner identifiers; and considering hemoglobin assessment when heavy or prolonged bleeding is reported. These are methodological priorities for future evaluation rather than interventions the present study showed to improve outcomes.

### 4.6. Strengths and Limitations

The principal limitations arise from the cross-sectional design, non-probability school-day sampling, and the structure of the source screening form. Temporal sequence and causality cannot be inferred, and no response rate or sampling fraction can be calculated. The analytic file did not retain exact screening months, a verified count of unique schools, urban/rural school classification, examiner identifiers, or device manufacturer/model/calibration metadata. Age was recorded only in completed years, preventing valid reconstruction of WHO BMI-for-age z-scores. Stress, nutrition, social wellbeing, reproductive status, menstrual flow, and pain were operational clinician-coded categories rather than validated instruments; menstrual blood loss was not quantitatively measured. Pubertal stage, gynecological age, socioeconomic indicators, physical activity, sleep, laboratory measurements (including hemoglobin/ferritin), and verified diagnoses were not available and may confound or modify the observed associations. Anthropometric measurements were examiner-obtained rather than self-reported, but inter-assessor technique and device variability could still introduce measurement error. Finally, the original spreadsheet contained a large block of exact duplicates and a small number of implausible or invalid values, indicating vulnerabilities in manual data consolidation.

Strengths include the large number of unique records, the inclusion of an understudied Central Asian adolescent population, direct rather than self-reported height and weight measurement, transparent deduplication and sensitivity analysis, prespecified composite outcomes, and modified Poisson regression with school-clustered robust standard errors. These strengths improve internal validity and precision but do not overcome limitations in the source measures or sampling design.

## 5. Conclusions

Among 4251 adolescent girls included in this school-based cross-sectional analysis, approximately one quarter had at least one recorded non-reference menstrual/reproductive indicator. Clinician-recorded stress was consistently associated with the primary composite outcome and with severe menstrual pain after multivariable adjustment and school-level clustering. The source-form body-mass categories showed marked age variation, but because they were based on fixed adult BMI cut points, they should not be interpreted as age-specific estimates of adolescent underweight or overweight.

The results should therefore be regarded as descriptive and hypothesis-generating. They highlight the need for future studies in this population to use WHO age-specific anthropometric classification, validated menstrual and psychosocial instruments, standardized measurement devices and school identifiers, relevant confounders such as socioeconomic status, physical activity and sleep, and prospective follow-up. Such work is required before conclusions can be drawn about causal relationships or the effectiveness of integrated school-health interventions.

## Figures and Tables

**Table 1:** Demographic and anthropometric characteristics of the analytic sample after removal of exact duplicate records.

Characteristic	*n*	Value
Age, 12 years	15	0.4%
Age, 13 years	369	8.7%
Age, 14 years	1206	28.4%
Age, 15 years	1188	27.9%
Age, 16 years	898	21.1%
Age, 17 years	495	11.6%
Age, 18 years	80	1.9%
Age, years	4251	15.03 ± 1.23; 15.00 (14.00–16.00)
Weight, kg	4247	53.37 ± 9.69; 52.00 (47.00–58.00)
Height, cm	4247	161.79 ± 6.23; 162.00 (158.00–166.00)
BMI, kg/m^2^	4247	20.36 ± 3.36; 19.90 (18.10–22.00)
Exploratory domain count	4251	0.83 ± 1.00; 1.00 (0.00–1.00)

BMI, body mass index. Continuous variables are reported as mean ± SD and median (IQR). Age-specific rows are reported as *n* (%).

**Table 2:** Recorded body-mass categories, Wilson 95% confidence intervals, and within-category BMI.

Recorded Category	*n*	%	95% CI	Median BMI (IQR), kg/m^2^
Underweight range (<18.5)	1263	29.71	28.36–31.10	17.3 (16.4–17.9)
Normal range (18.5–24.9)	2652	62.39	60.92–63.83	20.8 (19.6–22.2)
25.0–29.9	270	6.35	5.66–7.12	26.2 (25.5–27.6)
30.0–34.9	45	1.06	0.79–1.41	32.2 (30.9–33.3)
≥35.0	21	0.49	0.32–0.75	36.1 (35.3–38.4)

Percentages use the full analytic denominator (*n* = 4251); within-category median BMI is calculated among records that passed anthropometric plausibility checks. Categories follow adult cut points and are descriptive, not diagnostic, for adolescents.

**Table 3:** Prevalence of anthropometric, chronic, reproductive, menstrual, and psychosocial indicators.

Indicator	Events/*n*	Prevalence, %	Wilson 95% CI
Chronic disease recorded	54/4251	1.27	0.97–1.65
Underweight-range category	1263/4251	29.71	28.36–31.10
BMI ≥ 25 category	336/4251	7.90	7.13–8.75
BMI ≥ 30 category	66/4251	1.55	1.22–1.97
Non-reference reproductive status	93/4251	2.19	1.79–2.67
Non-reference menarche category	483/4251	11.36	10.44–12.35
Abnormal menstrual duration	291/4251	6.85	6.12–7.64
Abnormal menstrual flow	337/4251	7.93	7.15–8.78
Severe menstrual pain	292/4251	6.87	6.15–7.67
Amenorrhea/no-menses indicator	69/4251	1.62	1.28–2.05
Sexual activity reported	7/4250	0.16	0.08–0.34
Stress	292/4251	6.87	6.15–7.67
Unsatisfactory nutrition	87/4251	2.05	1.66–2.52
Unsatisfactory social wellbeing	15/4250	0.35	0.21–0.58

One sexual activity code and one social wellbeing code were outside the permitted range and were treated as missing, reducing the denominator to 4250 for these two indicators.

**Table 4:** Prevalence of selected outcomes by age group and tests of heterogeneity.

Outcome	12–13	14	15	16	17–18	χ^2^ (*p*)	V
Underweight range	38.3	37.8	26.6	26.5	18.4	96.34 (<0.001)	0.147
BMI ≥ 25	5.5	8.1	7.1	8.5	9.9	7.92 (0.095)	0.030
Non-reference reproductive status	1.3	2.5	2.0	2.3	2.3	2.18 (0.703)	0.000
Non-reference menarche	9.1	12.7	11.4	12.1	8.7	8.63 (0.071)	0.033
Any menstrual abnormality	21.9	22.8	23.8	27.8	23.0	9.48 (0.050)	0.036
Severe menstrual pain	4.2	4.2	6.9	9.4	10.3	36.54 (<0.001)	0.087
Stress	5.2	5.2	6.0	9.8	8.7	23.29 (<0.001)	0.067
Unsatisfactory nutrition	0.8	2.1	1.8	3.0	1.9	7.71 (0.103)	0.030

Values are percentages within each age group. χ^2^, chi-square statistic; V, bias-corrected Cramér’s V. Degrees of freedom = 4 for all tests.

**Table 5:** Adjusted prevalence ratios for menstrual and reproductive outcomes from modified Poisson regression with school-clustered robust standard errors.

Predictor	Any Menstrual Abnormality, aPR (95% CI)	*p*	Severe Menstrual Pain, aPR (95% CI)	*p*	Non-Reference Reproductive Status, aPR (95% CI)	*p*
Age, per year	1.03 (0.96–1.10)	0.488	1.26 (1.13–1.41)	<0.001	1.05 (0.84–1.31)	0.665
Underweight range	1.20 (1.02–1.42)	0.026	0.95 (0.74–1.22)	0.679	1.51 (0.98–2.31)	0.059
BMI ≥ 25	1.08 (0.89–1.32)	0.429	0.90 (0.59–1.38)	0.640	1.07 (0.57–2.02)	0.834
Chronic disease	1.43 (1.02–2.01)	0.040	2.13 (1.23–3.70)	0.007	0.77 (0.14–4.32)	0.765
Stress	2.05 (1.65–2.54)	<0.001	3.27 (2.39–4.46)	<0.001	2.78 (1.71–4.51)	<0.001
Unsatisfactory nutrition	1.64 (1.35–2.00)	<0.001	1.62 (0.95–2.76)	0.076	1.65 (0.76–3.55)	0.203

aPR, adjusted prevalence ratio; CI, confidence interval. Models included the predictors shown in the table. Outcome sample sizes/events: any menstrual abnormality, *n* = 4251/1024 events; severe menstrual pain, *n* = 4251/292 events; non-reference reproductive status, *n* = 4251/93 events.

**Table 6:** Adjusted prevalence ratios for psychosocial and anthropometric outcomes from modified Poisson regression with school-clustered robust standard errors.

Predictor	Stress, aPR (95% CI)	*p*	Underweight Range, aPR (95% CI)	*p*	BMI ≥ 25, aPR (95% CI)	*p*
Age, per year	1.20 (0.97–1.49)	0.099	0.83 (0.79–0.88)	<0.001	1.09 (1.00–1.19)	0.044
Underweight range	1.05 (0.78–1.41)	0.762	-	-	-	-
BMI ≥ 25	1.07 (0.66–1.72)	0.793	-	-	-	-
Chronic disease	0.96 (0.56–1.63)	0.881	1.15 (0.77–1.71)	0.504	1.11 (0.40–3.02)	0.845
Stress	-	-	1.03 (0.82–1.29)	0.778	1.06 (0.63–1.78)	0.824
Unsatisfactory nutrition	5.97 (3.79–9.40)	<0.001	1.48 (0.99–2.19)	0.054	1.41 (0.73–2.73)	0.312
Unsatisfactory social wellbeing	5.14 (2.91–9.07)	<0.001	-	-	-	-

aPR, adjusted prevalence ratio; CI, confidence interval. Outcome sample sizes/events: stress, *n* = 4250/292 events; underweight-range category, *n* = 4251/1263 events; BMI ≥ 25 category, *n* = 4251/336 events. Em dashes indicate covariates not entered in a given model.

**Table 7:** Sensitivity of key prevalence estimates to removal of exact duplicate records.

Indicator	Raw File (*n* = 5197)	After Deduplication (*n* = 4251)	Difference
Underweight range	29.29%	29.71%	+0.42 pp
BMI ≥ 25 category	7.91%	7.90%	−0.01 pp
Non-reference reproductive status	2.02%	2.19%	+0.17 pp
Stress	6.25%	6.87%	+0.62 pp
Unsatisfactory nutrition	1.92%	2.05%	+0.13 pp

## Data Availability

The data presented in this study are available on reasonable request from the corresponding author. The dataset is not publicly archived because it contains school-level information on minors.

## Abbreviations

The following abbreviations are used in this manuscript:

aPR	adjusted Prevalence Ratio
BMI	Body Mass Index
CI	Confidence Interval
IQR	Interquartile Range
SD	Standard Deviation
STROBE	Strengthening the Reporting of Observational Studies in Epidemiology

## Appendix A

**Table A1:** Coding scheme of the source screening form.

Field	Source Coding	Interpretive Basis/Caveat
Chronic disease	0 = no; 1 = yes	Source-form yes/no item; diagnosis text was incomplete in some records.
Body-mass category	0 = normal; 1–3 = increasing high-BMI categories; 4 = mass deficit	Operational adult cut points in source form; not a pediatric diagnosis. WHO BMI-for-age is preferred [25,26].
Reproductive health status	0 = corresponds to age; 1 = advanced; 2 = delayed; 3 = uncertain	Program-specific clinician-coded category; not a validated reproductive-development scale.
Age at menarche	0 = 11–13 years; 1 = 9–10 years; 2 = 14–15 years; 3 = after 15 years; 4 = no menses	Operational source categories. Adolescent menstrual interpretation referenced to clinical guidance [7].
Menstrual duration	0 = 3–7 days; 1 = under 3 days; 2 = over 7 days; 3 = amenorrhea	Operational categories; not a diagnostic instrument [7].
Menstrual flow	0 = moderate; 1 = scanty; 2 = heavy; 3 = no menses	Qualitative clinician-coded categories; blood loss volume was not measured [7,20].
Menstrual pain	0 = moderate; 1 = severe; 2 = no menses	Clinician-coded moderate/severe/no-menses item; no validated pain scale was used [7,10].
Sexual activity	0 = no; 1 = yes	Single self-report/clinician-recorded binary item; subject to social desirability bias.
Stress	0 = no; 1 = yes	Single clinician-coded binary item; not a validated stress scale.
Nutrition	0 = satisfactory; 1 = unsatisfactory	Single clinician-coded binary item; no dietary instrument or laboratory validation.
Social wellbeing	0 = satisfactory; 1 = unsatisfactory	Single clinician-coded binary item; not a validated psychosocial scale.
